# Factors associated with patients’ and companions’ satisfaction with a hospital emergency department: A descriptive, cross‐sectional study

**DOI:** 10.1002/nop2.261

**Published:** 2019-03-28

**Authors:** Aurora Fontova-Almató, Rosa Suñer‐Soler, Dolors Juvinyà‐Canal

**Affiliations:** ^1^ Emergency Department Fundació Salut Empordà Girona Spain; ^2^ Faculty of Nursing University of Girona Girona Spain; ^3^ Health and Healthcare Research Group University of Girona Girona Spain

**Keywords:** emergency department, emergency nursing, health promotion, patient satisfaction, quality of care

## Abstract

**Aim:**

The aim was to study the level of satisfaction with an emergency department and to identify the factors associated with satisfaction.

**Design:**

This research consisted of a descriptive, cross‐sectional study.

**Methods:**

The study population was composed of patients and companions who visited the emergency department during a three‐month period. The patients were selected randomly from the register of visits to the emergency department during the period of study. Sociodemographic variables and variables related to the attention received were studied through questionnaires (response rate 33%).

**Results:**

Older age was associated with greater satisfaction (*p* = 0.002), as was female sex (*p* = 0.013) and greater optimism (*p* < 0.001). Greater control of pain was a factor associated with satisfaction (*p* = 0.033), as was the perception of a shorter waiting time before the medical visit (*p* < 0.001).

## 1 INTRODUCTION

Satisfaction with emergency departments affects overall satisfaction with hospitalization (Kardanmoghadam et al., 2015), and the evaluation of patient and companion satisfaction is a measurement of the quality of care in emergency departments (Danielsen et al., 2010; Granado de la Orden et al., 2011; Jennings, Lee, Chao, & Keating, 2009; Magaret, Clark, Warden, Magnusson, & Hedges, 2002; Soleimanpour et al., 2011; Welch, 2010).

Patient satisfaction refers to the subjective experience of the user of the health system (Boxer & Boxer Goldfarb, 2009). A satisfied patient better adheres to treatment, makes fewer malpractice complaints and is more willing to return to the service than otherwise (Boxer & Boxer Goldfarb, 2009). When a person goes to an emergency department, he or she is normally accompanied by a family member, friend or carer, who in many cases will give emotional support and will be able to give relevant information about the functional status of the patient (Ekwall, Gerdtz, & Manias, 2008; Nikki, Lepistö, & Paavilainen, 2012). Friends and family members play an important role in the care and well‐being of the patient in the emergency department (Gordon, Sheppard, & Anaf, 2010), and the experience in the department affects companions' perception of the quality of care (Cooke, Watt, Wertzler, & Quan, 2006; Ekwall et al., 2008; Parra Hidalgo, Calle Urra, Ramón Esparza, Peiró Moreno, & Meneu de Guillerna, 2012).

In a review of the literature about patient and companion satisfaction with emergency departments, where studies of patient characteristics and perceived waiting times particularly stand out, older patients are found to be more satisfied than younger patients (Crow et al., 2002; Danielsen et al., 2010; Ekwall et al., 2008; Quintana et al., 2006; Welch, 2010) and the perceived waiting time is particularly important for user satisfaction (Boudreaux & O'Hea, 2004; Brown, Sandoval, Levinton, & Blackstien‐Hirsch, 2005; Taylor & Benger, 2004). Information and communication in the emergency department were also determining factors in patient satisfaction (Boudreaux & O'Hea, 2004; Nairn, Whotton, Marshal, Roberts, & Swann, 2004), as well as pain, with an association being found between the easing of pain and satisfaction (Muntlin, Gunningberg, & Carlsson, 2006; Welch, 2010). Furthermore, some variables related to patient satisfaction with emergency departments, such as information and communication, perceived waiting time and older age, coincide with what is found in the case of companions (Ekwall et al., 2008; Ekwall, Gerdtz, & Manias, 2009; Kristensson & Ekwall, 2008; Magaret et al., 2002; Morales‐Guijarro, Nogales‐Cortés, & Pérez‐Tirado, 2011; Pérez‐Tirado, Hernández‐Blanco, Nogales‐Cortés, & Sánchez‐Sánchez, 2010). Other factors, such as being able to accompany the patient or participate in his or her care, also have an influence on companions (Nairn et al., 2004; Soleimanpour et al., 2011).

Based on the above, we can affirm that the satisfaction of patients and companions with emergency departments is a measure of quality of care and a factor that influences both the choice of the service by the user and adherence to the treatment prescribed. However, we were unaware of the profile of the most satisfied users and companions in our setting and so we proposed the following objectives:

- To study the level of satisfaction of patients and companions with an emergency department.
- To identify the factors associated with the satisfaction level of patients and companions with an emergency department.
- To determine the profile of the most satisfied users of the emergency department.

## 2 METHODS

### 2.1 Design

The research consisted of a descriptive, cross‐sectional study of the satisfaction of patients and companions. The participants voluntarily accepted to participate in this study, which was conducted in the emergency department of a general hospital. The field was undertaken using questionnaires from October to December 2012.

### 2.2 Study settings

This study was performed at a general hospital that attended 65,071 emergencies, a mean average of 178 visits per day.

The study population was composed of patients and companions who visited the emergency department of a general hospital from October to December 2012. The sampling technique was probabilistic, involving a random selection of patients from four tables of random numbers from the daily register of visits to the emergency department during the 3‐month period.

### 2.3 Participants

All patients over 18 years of age were included. Patients residing outside the Spanish territory were excluded from the study, as were those who left the service without being visited, those who needed only nursing care and hospital workers who were seen at the service. When a patient who was selected died during the medical event, a letter was sent to family members expressing condolences and explaining the aim of the study.

The sample size was calculated considering a 10‐point difference with regard to the results obtained in the study of González et al. (2008); thus to obtain a power of 80% with a significance level of 0.05, 194 patients and companions would be needed, calculating response losses of 40%.

### 2.4 Measures

A satisfaction questionnaire was sent by ordinary post to the selected patients. At the beginning of the questionnaire, there was an item to identify whether the person responding was a patient or a companion. When the questionnaire was completed together by both a patient and a companion, the answers were treated as though they were of the patient.

A letter was sent to the home address of all the patients selected within the 10 days following the visit; it contained an information sheet about the study, a letter of informed consent to be signed, the questionnaire and a pre‐paid envelope to return the questionnaire to the hospital. One month after the first mailing, a reminder letter was sent to all those who had not yet replied to the questionnaire. Due to the low response rate, selected patients were contacted via telephone to determine if a third mailing would be necessary. All of those who agreed to reply were again sent a questionnaire with a pre‐paid envelope.

The first variables studied were sociodemographic: age, sex, civil status, educational level and occupation. Questions on the following were asked: overall satisfaction with the visit to the emergency department (on a scale of 0–10, where 0 was the worst possible score and 10 the best possible score); pain (were you in pain when you visited the emergency department? with two possible responses: yes or no) and whether this pain was adequately controlled (answered via a Likert scale: no, not at all; slightly but not completely; quite a lot but not completely; or yes, completely); the waiting time to be seen for the first time by a nurse (in minutes); the perception of this time (answered via a Likert scale: very short, short, acceptable, long or very long); the waiting time to be seen by a doctor (in minutes); the perception of the waiting time for the medical visit (answered via a Likert scale: very short, short, acceptable, long or extremely long); information on the approximate waiting time (whether or not information was received); and whether or not they would recommend the department to a family member or friend (yes or no). Additionally, the perceived level of optimism was studied (on a scale from 0–10, where 0 was the worst score possible and 10 the best).

To study the factors associated with satisfaction, the numerical global satisfaction variable was recoded into two categories: “less satisfied,” which included those users who gave a score of between 0–7 and “more satisfied,” which included those who gave a score of between 8–10 points. The pain control variable was also recoded as “pain controlled,” for those who had referred to their pain being sufficiently or completely controlled and “pain not controlled” for those referred to their pain having been little or not controlled.

The satisfaction questionnaire validated by González et al. (2005) was administered, with the authors' previous consent. This questionnaire consists of 34 questions grouped into six dimensions: information and medical care, nursing care, comfort, visiting, privacy and cleanliness. Cronbach's alpha coefficient results were above 0.7 for all dimensions except privacy, where a coefficient of 0.60 was obtained. In our study, Cronbach's alpha coefficient results were above 0.8 for information and medical care and nursing care, except comfort, visiting, privacy and cleanliness where a coefficient under 0.65 was obtained.

Data from the clinical history were also gathered to perform analysis with the responses of the patients and companions: date of birth, date of the visit, time of arrival at the emergency department, electronically recorded time of the nursing triage and electronically recorded time at which the doctor assigned the date and time of release from the department. In the case of companions, data related to the patient's visit were used.

### 2.5 Analysis

IBM^®^ SPSS Statistics^®^ version 19 was used to analyse the data.

The numerical variables are described by mean and standard deviation or by median and interquartile range. To study the association between the variables that met the criteria of normality, Student's *t* test was used to compare quantitative and categorical variables, the Pearson's correlation was used to compare quantitative variables and the chi‐squared test was used for categorical variables. For variables that did not meet the criteria of normality, the Mann–Whitney *U* test and the Kruskal–Wallis non‐parametric test were used to compare quantitative and categorical variables and the Spearman's rho test was used for quantitative variables. A binary regression model was run from factors associated with patients' and companions' satisfaction with the emergency department adjusting for age, sex and level of optimism. The level of significance was taken as *p* < 0.05.

### 2.6 Ethics

All the participants who responded to the questionnaire also returned the signed informed consent. This project respects the Helsinki Declaration of the World Medical Association. The study was approved on 1 August 2012 by the ethical committee of the hospital before beginning the study.

## 3 RESULTS

A total of 15,273 patients were attended to, from whom, 1,526 patients were randomly selected (864 patients were included and 662 were excluded). A total of 285 responses were received (response rate 33.0%); of which, 221 (77.5%) corresponded to patients, 62 (21.8%) to companions and 2 (0.7%) to respondents who failed to identify themselves as either a patient or companion. The mean age of the respondents was 54.6 years (*SD* = 18.3), and 53.6% were women. The sociodemographic data of the patients and companions are presented in Table 1.

**Table 1:** Sociodemographic characteristics and studied variables of patients and companions

	Patients (*N*) %	Companions (*N*) %	*p*
Sex			
Female	108 (48.9)	42 (73.7)	0.001
Civil status			
Married/couple	149 (68.0)	43 (70.5)	0.381
Single	27 (12.3)	3 (4.9)	
Widow/widower	28 (12.8)	10 (16.4)	
Divorced	15 (6.8)	5 (8.2)	
Educational level			
Without studies	45 (20.8)	13 (22.0)	0.837
Primary	98 (45.4)	26 (44.1)	
Secondary	51 (23.6)	16 (27.1)	
University	22 (10.2)	4 (6.8)	
Perception of control of pain			
Not controlled at all	19 (10.4)	5 (10.0)	0.821
Slightly but not completely	32 (17.6)	11 (22.0)	
Quite a lot but not complete	66 (36.3)	15 (30.0)	
Completely controlled	65 (35.7)	19 (38.0)	
Perceived waiting time for triage			
Very short	47 (21.6)	15 (24.2)	0.648
Short	38 (17.4)	10 (16.1)	
Adequate	103 (47.2)	30 (48.4)	
Long	25 (11.5)	4 (6.5)	
Extremely long	5 (2.3)	3 (4.8)	
Perceived waiting time for medical attention			
Very short	32 (14.7)	10 (16.1)	0.485
Short	3 (15.7)	9 (14.5)	
Adequate	83 (38.2)	29 (46.8)	
Long	54 (24.9)	9 (14.5)	
Extremely long	14 (6.5)	5 (8.1)	
Information about the waiting time			
No	172 (79.3)	45 (75.0)	0.478
Would you recommend the department?			
Yes	187 (87.4)	56 (93.3)	0.198
Age	221 55.3 (18.6); 55.0 [29]	36 50.6 (15.2); 53.5 [18]	0.150
Level of optimism	205 7.2 (2.0); 8.0 [3]	56 7.1 (2.0); 7.0 [3]	0.677
Satisfaction with visit	205 7.6 (2.2); 8.0 [2]	56 7.5 (2.1); 8.0 [3]	0.693

Note

Qualitative variables are expressed as an absolute frequency and with percentages in brackets. Quantitative variables are expressed as the average (standard deviation) and median [interquartile range].

For patients and companions, the mean score for subjective overall satisfaction with the visit (*N* = 273) was 7.6 (*SD* = 2.2) [median = 8; IQR = 2]. The patients (*N* = 214) and companions (*N* = 57) assigned a mean satisfaction score of 7.6 (*SD* = 2.2) [median = 8; IQR = 2] and 7.5 (*SD* = 2.1) [median = 8; IQR = 3], respectively, without significant differences between the two scores (Mann–Whitney *U* test, *p* = 0.390).

The results of the individual dimensions of the satisfaction scale taken separately were as follows: for the dimension of medical care, a mean score of 84 (*SD* = 17.2) [median = 89.6; IQR = 24.1], for nursing care 79.7 (*SD* = 19.9) [median = 84.2; IQR = 26.3], for comfort in the department 65.7 (*SD* = 19.1) [median = 64.7; IQR = 23.5], for visiting 78.3 (*SD* = 11.7) [median = 81.8; IQR = 9.1], for privacy 79.6 (*SD* = 30.3) [median = 100; IQR = 25] and for cleanliness 91.3 (*SD* = 14.1) [median = 100; IQR = 16.7].

Analysing the association between subjective overall satisfaction and the age of the participants shows that older patients scored higher than younger patients with respect to overall satisfaction with the visit (*r* = 0.236; *p *< 0.001) (Pearson's correlation).

The presence of pain was compared with overall satisfaction. Patients with pain gave a mean satisfaction score of 7.5 (*SD* = 2.3) [median = 8; IQR = 2], whereas those who were not in pain gave a mean score of 7.8 (*SD* = 1.6) [median = 8; IQR = 2] (Mann–Whitney *U* test, *p *= 0.898). On the other hand, studying the association of control of pain and overall satisfaction shows that people whose pain was better controlled gave higher scores for overall satisfaction with the visit (Kruskal–Wallis test, *p* < 0.001).

The perceived waiting time for nursing triage was compared with subjective overall satisfaction. It was found that patients who perceived that they had waited for a very short or short period of time gave higher scores for subjective satisfaction than did those who perceived that they had waited a long or extremely long time (Kruskal–Wallis test, *p *< 0.001). The relationship between the perceived waiting time for the medical visit and subjective overall satisfaction was also studied; patients who had the perception of having waited for a short or for a very short period of time gave higher scores for subjective satisfaction than did those who had the perception of having waited for a long time or for an extremely long time (Kruskal–Wallis test, *p *< 0.001).

Furthermore, the association between information about the waiting time and satisfaction was analysed. Patients and companions who were informed about the waiting time gave a mean score of 8.4 (*SD* = 1.8) [median = 9; IQR = 3], whereas those who were not informed gave a mean score of 7.4 (*SD* = 2.2) [median = 8; IQR = 3], (Mann–Whitney *U* test, *p* = 0.001).

A total of 88.8% of the respondents would recommend the emergency department to a family member or friend. Analysing the possibility of recommending the department via subjective overall satisfaction shows that those who would recommend the department gave a mean score of 8.0 (*SD* = 1.7) [median = 8; IQR = 2] for overall satisfaction and that those who would not recommend it gave a mean of 4.6 (*SD* = 2.9) [median = 4; IQR = 5], (Mann–Whitney *U* test, *p *< 0.001).

The level of perceived optimism of patients and companions had a mean score of 7.2 (*SD* = 2.0) [median = 8; IQR = 3].

Analysing the relationship between the level of optimism and subjective satisfaction with the visit shows a positive correlation between the two variables, with greater levels of optimism being found to correspond to greater overall satisfaction (Spearman's rho test; *ρ* = 0.502; *p *< 0.001).

Table 2 shows the profile of the most and least satisfied users from the variables studied. The more satisfied users had a greater mean age (*t*‐Student test; *p *< 0.001) than the less satisfied users, and women were found to be in more satisfied group (*χ* ^2^ = 4.98; *p *= 0.026). Observably, people who scored better in optimism were in the most satisfied group (Mann–Whitney *U* test; *p *< 0.001). Furthermore, people who had better controlled pain were found to be in the group of the most satisfied users (80.9% vs. 57%) (*χ* ^2^ = 15.12; *p *< 0.001).

**Table 2:** Characteristics of patients and companions by level of satisfaction

	Less satisfied (0–7)	More satisfied (8–10)	*p*
*N* (%)	108 (39.6)	165 (60.4)	
Age (average and *SD*)	49.4 (18.1)	58.12 (17.6)	<0.001
Sex (% women)	45.3	59.1	0.026
Level of optimism (average and *SD*; median and IQR)	6.21 (2.1); 7 (3)	7.9 (1.7); 8 (2)	<0.001
Control of pain (% sufficiently‐completely controlled)	57.0	80.9	<0.001
Perceived waiting time for triage (% very short‐short)	23.4	49.4	<0.001
Perceived waiting time until medical visit (% very short‐short)	16.8	38.3	<0.001
Information about the approximate waiting time (% no)	86.9	75.6	0.023
Would you recommend the emergency department? (% yes)	77	95.7	<0.001

Regarding the perceived waiting times, people who perceived that the waiting time for the visit of the triage nurse was very short or short were found to be in the group of the most satisfied users (49.4% vs. 23.4%) (*χ* ^2^ = 28.87; *p *< 0.001); the same occurred with the perceived waiting time for the medical visit, where those who considered this time to be very short or short were also among the group of the most satisfied users (38.3% vs. 16.8%) (*χ* ^2^ = 34.14; *p *< 0.001). People who were not informed about the approximate waiting time for the medical visit were among the least satisfied (86.9% vs. 75.6%) (*χ* ^2^ = 5.14; *p *= 0.023), and people who would recommend the emergency department to a family member or friend were among the group of the most satisfied users (95.7% vs. 77%) (*χ* ^2^ = 21.64; *p *< 0.001).

Table 3 shows the results of the binary logistic regression model, which reveals that age, sex and optimism were all factors associated with satisfaction. Age was significantly associated with greater satisfaction (OR = 1.035; CI 95% [1.01–1.06]; *p *= 0.002), as was the female sex (OR = 0.399; CI 95% [0.19–0.82]; *p *= 0.013). The level of optimism was also associated with user satisfaction, with the most optimistic people being the most satisfied (OR = 1.679; CI 95% [1.33–2.11]; *p *< 0.001).

**Table 3:** Factors associated with the satisfaction of patients and companions^a^

Variable	*B*	ET	Wald	*p*	OR	CI 95%	
						Lower	Higher
Age	0.034	0.011	9.783	0.002	1.035	1.013	1.057
Sex	−0.920	0.371	6.146	0.013	0.399	0.193	0.825
Optimism	0.518	0.117	19.615	0.000	1.679	1.335	2.111
Control of pain	−0.873	0.409	4.544	0.033	0.418	0.187	0.932
Perceived time for medical visit			14.512	0.001			
Perceived time for medical visit 1	1.697	0.479	12.569	0.000	5.456	2.136	13.941
Perceived time for medical visit 2	1.254	0.438	8.214	0.004	3.506	1.487	8.266

Notes

Perceived time for medical visit: long or extremely long; Perceived time for medical visit 1: very short or short; Perceived time for medical visit 2: acceptable; Sex reference variable inserted into the model was male.

a Binary logistic regression.

Control of pain was a factor associated with satisfaction and greater control of pain was associated with greater satisfaction (OR = 0.418; CI 95% [0.19–0.93]; *p *= 0.033). The perceived waiting time was also associated with satisfaction, with those who perceived that the waiting time for the medical visit was very short being among the most satisfied (OR = 5.456; CI 95% [2.14–13.94]; *p *< 0.001).

## 4 DISCUSSION

The present study has identified the factors associated with the patients' and companions' satisfaction with the emergency department of a Spanish general hospital.

The chosen method for the questionnaire, a mailing with a pre‐paid envelope, achieved a 33% response rate, which was similar to that of other studies (González‐Valentín, Padín López, & de Ramón Garrido, 2005; Pujiula‐Masó et al., 2006).

Subjective overall satisfaction with the visit to the emergency department was high, with a mean score of 7.6, which is in line with the scores obtained in similar studies (Gea, Hernán‐García, Jiménez‐Martín, & Cabrera, 2001; Parra Hidalgo et al., 2012). The dimension on the satisfaction scale that scored best was cleanliness, followed by information and medical care; in contrast, the worst scores were for comfort. A comparison with the results found in the literature shows that the dimensions of information and medical care and nursing care were better valued than in the study of González et al. (2008), where three of the four hospitals surveyed obtained lower scores in this dimension. On the other hand, with regard to privacy, the scores given by the patients and companions in our study were lower than those of these authors.

Older patients were found to be more satisfied, coinciding with other authors (Crow et al., 2002; Quintana et al., 2006). Additionally, women were found to be more likely to belong to the group of more satisfied users, unlike in previous studies, which have not found sex to have any relationship with satisfaction (Crow et al., 2002).

In our study, more than eight out of ten of the patients surveyed referred to pain when they visited the emergency department and in most cases, the pain was controlled completely or sufficiently. This result is similar to Kamali, Jain, Jain, and Schneider (2013), who found that more than half of all patients referred to having had some type of pain on arrival at the emergency department. In the present study, satisfaction with the service was not associated with the presence of pain but, rather, with the control of pain. Patients whose pain was controlled sufficiently or completely were among the group of most satisfied users, which is in line with previous studies (Byczkowski et al., 2013; Soleimanpour et al., 2011; Welch, 2010). Similarly, Marco, Davis, Chang, Mann, and Olson (2015) observed that the treatment of pain was a factor of dissatisfaction in a study carried out in a hospital emergency department. Muntlin et al. (2006) also observed that patients were not satisfied with the pain relief and that pain continues to be undertreated in emergency departments; thus, they recommend that pain should be treated individually. Furthermore, there are authors who consider that the implementation of a plan for pain management can be effective in improving patient satisfaction (Nairn et al., 2004; Welch, 2010).

In our study, the perception of the waiting time was one of the factors that most influenced the subjective overall satisfaction of users in terms of both nursing care and the medical visit. Similarly, in a study undertaken in an emergency department, patients who referred to having waited less time were more satisfied than those who had waited longer (Mercer, Hernandez‐Boussard, Mahadevan, & Strehlow, 2014). A long or extremely long perceived waiting time has been shown to be negatively associated with satisfaction and users who referred to this aspect were the least satisfied, which is in line with earlier findings (Bos, Van Stel, Schrijvers, & Sturms, 2015; Brown et al., 2005; Nairn et al., 2004).

Being informed of the approximate waiting time also influences users’ satisfaction. The present results show that users who were informed about the approximate waiting time gave better scores for satisfaction with the service. In an investigation in an emergency department, Kington and Short (2010) observed that those who were surveyed asked for greater information about the waiting time. Similarly, Burström, Starrin, Engström, and Thulesius (2013) observed that giving information about the approximate waiting time and the attitudes of the staff were important aspects with regard to patient satisfaction. Along the same lines, in a literature review, Innes, Jackson, Plummer, and Elliott (2015) highlighted the importance of communicating the waiting times in emergency departments.

Patient satisfaction must include, along with overall satisfaction with the service, the probability that the patient will recommend the service to others and the willingness to return to the service (Welch, 2010). As in earlier studies (Boudreaux & O'Hea, 2004; Parker & Marco, 2014; Soleimanpour et al., 2011), we observed that user satisfaction is a key component in the choice and recommendation of an emergency service. On the other hand, user dissatisfaction may affect the viability of health institutions (Boudreaux & O'Hea, 2004) and influence the perception that users have of a hospital (Broadwater‐Hollifield et al., 2014; Wagner, 2014).

Regarding optimism, the most satisfied users scored higher for optimism. Costello et al. (2008) observed that pessimistic patients gave lower scores for their level of satisfaction with the care received.

Identifying the factors associated with user satisfaction makes it possible to define strategies and actions to improve the quality of the emergency department. It is especially important to improve the control of pain in the emergency department and to reduce the real and perceived waiting times as well as to improve the supply of information about these times.

### 4.1 Limitations

The possible limitations of the present study are related to the methodology employed; the cross‐sectional design does not allow causality to be established. Furthermore, we have no way of knowing the motivations of those who left the service without being visited and it is likely that these may include dissatisfaction with the service. One of the strengths of this investigation is the inclusion of companions in the patient satisfaction study.

## 5 CONCLUSION

The level of satisfaction with the emergency department was high, and the factors associated with the satisfaction of patients and companions were age, sex, optimism, control of pain and the perceived waiting time before the medical visit.

The most satisfied users were those who were older, women, patients whose pain was better controlled and those who perceived shorter waiting times and who were informed of an approximate waiting time.
